# Transurethral MR-HIFU for the treatment of localized prostate cancer

**DOI:** 10.1186/2050-5736-3-S1-O59

**Published:** 2015-06-30

**Authors:** Rajiv Chopra

**Affiliations:** 1 University of Texas Southwestern Medical Center, Dallas, Texas, United States

## Background/introduction

Transurethral MR-HIFU involves the delivery of high-intensity ultrasound energy for targeted coagulation of prostate tissue under real-time MRI temperature control.

## Methods

In contrast to transrectal approaches, transurethral ultrasound applicators are simple in construction and designed for single use. Delivery of ultrasound from within the prostate gland avoids passing energy through sensitive structures, and enables rapid tissue ablation. Tissue is coagulated radially outwards from the urethra, with the depth of treatment controlled based on the temperatures measured with MR thermometry. One of the challenges with transurethral HIFU is generating coagulation to the boundary of the prostate gland where tumors are often located.

## Results and conclusions

Transurethral HIFU has been under development for over two decades with a variety of transducer designs. Recently, this technology has been translated into human studies, and is being developed as a commercial treatment for localized prostate cancer. Phase 0 are complete and Phase 1 trials are underway. This talk will provide a review of the technology, its development, clinical translation, and unique characteristics. Current challenges and opportunities for future development will also be discussed.

